# Aberrant Hippocampal Development in Early-onset Mental Disorders and Promising Interventions: Evidence from a Translational Study

**DOI:** 10.1007/s12264-023-01162-2

**Published:** 2023-12-23

**Authors:** Jingyu Yang, Huiling Guo, Aoling Cai, Junjie Zheng, Juan Liu, Yao Xiao, Sihua Ren, Dandan Sun, Jia Duan, Tongtong Zhao, Jingwei Tang, Xizhe Zhang, Rongxin Zhu, Jie Wang, Fei Wang

**Affiliations:** 1 Early Intervention Unit, Department of Psychiatry, Affiliated Nanjing Brain Hospital, Nanjing Medical University, Nanjing, 210029 China; 2 School of Biomedical Engineering and Informatics, Nanjing, Medical University, Nanjing, 211166 China; 3 The Affiliated Changzhou Second People’s Hospital of Nanjing Medical University, Changzhou Second People’s Hospital, Changzhou Medical Center, Nanjing Medical University, Changzhou, 213004 China; 4 Department of Radiology, First Hospital of China Medical University, Shenyang, 110002 China; 5 Department of Cardiac Function, The People’s Hospital of China Medical University and the People’s Hospital of Liaoning Province, Shenyang, 110067 China; 6 Key Laboratory of Magnetic Resonance in Biological Systems, State Key Laboratory of Magnetic Resonance and Atomic and Molecular Physics, National Center for Magnetic Resonance in Wuhan, Wuhan Institute of Physics and Mathematics, Innovation Academy for Precision Measurement Science and Technology, Chinese Academy of Sciences-Wuhan National Laboratory for Optoelectronics, Wuhan, 430064 China; 7 Institute of Neuroscience and Brain Diseases; Xiangyang Central Hospital, Affiliated Hospital of Hubei University of Arts and Science, Xiangyang, 441021 China; 8 Functional Brain Imaging Institute of Nanjing Medical University, Nanjing, 210029 China

**Keywords:** Early-onset, Mental disorder, Neurodevelopment, Animal model, Hippocampus, Repetitive transcranial magnetic stimulation

## Abstract

Early-onset mental disorders are associated with disrupted neurodevelopmental processes during adolescence. The methylazoxymethanol acetate (MAM) animal model, in which disruption in neurodevelopmental processes is induced, mimics the abnormal neurodevelopment associated with early-onset mental disorders from an etiological perspective. We conducted longitudinal structural magnetic resonance imaging (MRI) scans during childhood, adolescence, and adulthood in MAM rats to identify specific brain regions and critical windows for intervention. Then, the effect of repetitive transcranial magnetic stimulation (rTMS) intervention on the target brain region during the critical window was investigated. In addition, the efficacy of this intervention paradigm was tested in a group of adolescent patients with early-onset mental disorders (diagnosed with major depressive disorder or bipolar disorder) to evaluate its clinical translational potential. The results demonstrated that, compared to the control group, the MAM rats exhibited significantly lower striatal volume from childhood to adulthood (all *P* <0.001). In contrast, the volume of the hippocampus did not show significant differences during childhood (*P* >0.05) but was significantly lower than the control group from adolescence to adulthood (both *P* <0.001). Subsequently, rTMS was applied to the occipital cortex, which is anatomically connected to the hippocampus, in the MAM models during adolescence. The MAM-rTMS group showed a significant increase in hippocampal volume compared to the MAM-sham group (*P* <0.01), while the volume of the striatum remained unchanged (*P* >0.05). In the clinical trial, adolescents with early-onset mental disorders showed a significant increase in hippocampal volume after rTMS treatment compared to baseline (*P* <0.01), and these volumetric changes were associated with improvement in depressive symptoms (*r* = − 0.524, *P* = 0.018). These findings highlight the potential of targeting aberrant hippocampal development during adolescence as a viable intervention for early-onset mental disorders with neurodevelopmental etiology as well as the promise of rTMS as a therapeutic approach for mitigating aberrant neurodevelopmental processes and alleviating clinical symptoms.

**Supplementary Information:**

The online version contains supplementary material available at 10.1007/s12264-023-01162-2.

## Introduction

Most mental disorders first begin before the age of 24 years [1, 2], and the development of these disorders is thought to be caused by disruptions in the typical development of the adolescent brain [3]. Adolescence is a period characterized by physiological, psychological, and brain development [4, 5]. Therefore, early-onset mental disorders might have long-term effects, suggesting that adolescence is not only a critical period for early diagnosis but also for early intervention [6]. In addition, the current diagnostic framework, for conditions such as major depressive disorder (MDD), bipolar disorder (BD), or schizophrenia, which relies on symptomology, shows high symptom overlap between different diagnoses and is not well-suited for the early identification of mental disorders, especially in adolescents [7, 8]. The lack of objective biological markers for these disorders further complicates early identification and intervention [9]. Therefore, the identification of early biomarkers of early-onset mental disorders is crucial for early diagnosis and early intervention.

Previous research has indicated that early-onset mental disorders, whether mood disorders or psychotic disorders, are associated with the alteration of normal neurodevelopmental trajectories [10, 11]. Interference with the sensitive early stages of neurodevelopment can have a profound impact on the subsequent developmental process [12]. Studies have shown that early-onset mental disorders exhibit similar reductions in intracranial volume, regardless of the specific diagnosis, such as cortical thinning and reduced volume in the hippocampus, thalamus, amygdala, basal ganglia, and striatum [13–15]. Importantly, these structural changes have been observed early in the course of the disorders [16] and have been found to predict changes in clinical symptoms [17]. Therefore, structural developmental abnormalities may serve as early imaging markers of early-onset mental disorders. In longitudinal human studies of neurodevelopment, it can be challenging to capture the dynamic nature of disease progression, but animal models can provide insight into these processes and help to understand the biological basis of early-onset mental disorders.

The methylazoxymethanol acetate (MAM) animal model, which induces abnormal neurodevelopmental processes during the prenatal period and leads to widespread cortical structural changes and cognitive impairments [18–21], was originally applied to explore the abnormal neurodevelopmental processes associated with schizophrenia [22, 23]. In this study, we used the MAM model to simulate the neurodevelopmental processes of early-onset mood disorders (MDD and BD) from an etiological perspective. Our previous research has demonstrated that animal models with similar etiology and clinical subtypes exhibit similar neuroimaging patterns, while those with different etiology display distinct neuroimaging patterns [24]. Therefore, neuroimaging has the potential to serve as an objective phenotype that links animal models and clinical populations with similar etiologic mechanisms, making it a potential biological marker for cross-species translational research [25, 26]. In this study, we aimed to redefine clinical subtypes using a homogeneous animal model based on a single etiological factor to simulate the neurodevelopmental trajectory of early-onset mental disorders. Using neuroimaging as a biological phenotype, we investigated brain structural changes at different developmental stages to explore critical windows and targets for early intervention.

Repetitive transcranial magnetic stimulation (rTMS) is a widely used tool in physical therapy for treating mental disorders [27]. However, the efficacy of rTMS in adolescent patients is uncertain at present, possibly due to a lack of specificity of the treatment target [28–31]. Most studies currently use functional magnetic resonance imaging (fMRI) to identify abnormal brain regions and target these sites to explore the effect of rTMS intervention [32]. However, few studies have used structural MRI to guide rTMS intervention and evaluate its effectiveness. It has been suggested that rTMS may influence brain structure by regulating neuronal plasticity [33, 34]. Since adolescence is a crucial period for the development of brain plasticity, we speculated that early rTMS intervention may impact the brain development process through the modulation of brain plasticity. Therefore, using animal models to explore abnormal biological characteristics and targeting the critical windows with rTMS to further explore whether the abnormal brain development trajectory can be returned to normal, which may improve its effectiveness in treating early-onset adolescent patients with mental disorders.

In this study, we aim to evaluate the neurodevelopmental processes of early-onset mental disorders through longitudinal MRI recordings on the MAM animal model. Additionally, the rTMS intervention effects on the critical windows of disease progression were investigated in both the MAM animal model and patients with early-onset mental disorders. This cross-species research would enhance our comprehension of neurodevelopmental processes and critical windows for intervention.

## Materials and Methods

### Overview of the Experimental Design

**Fig. 1:**
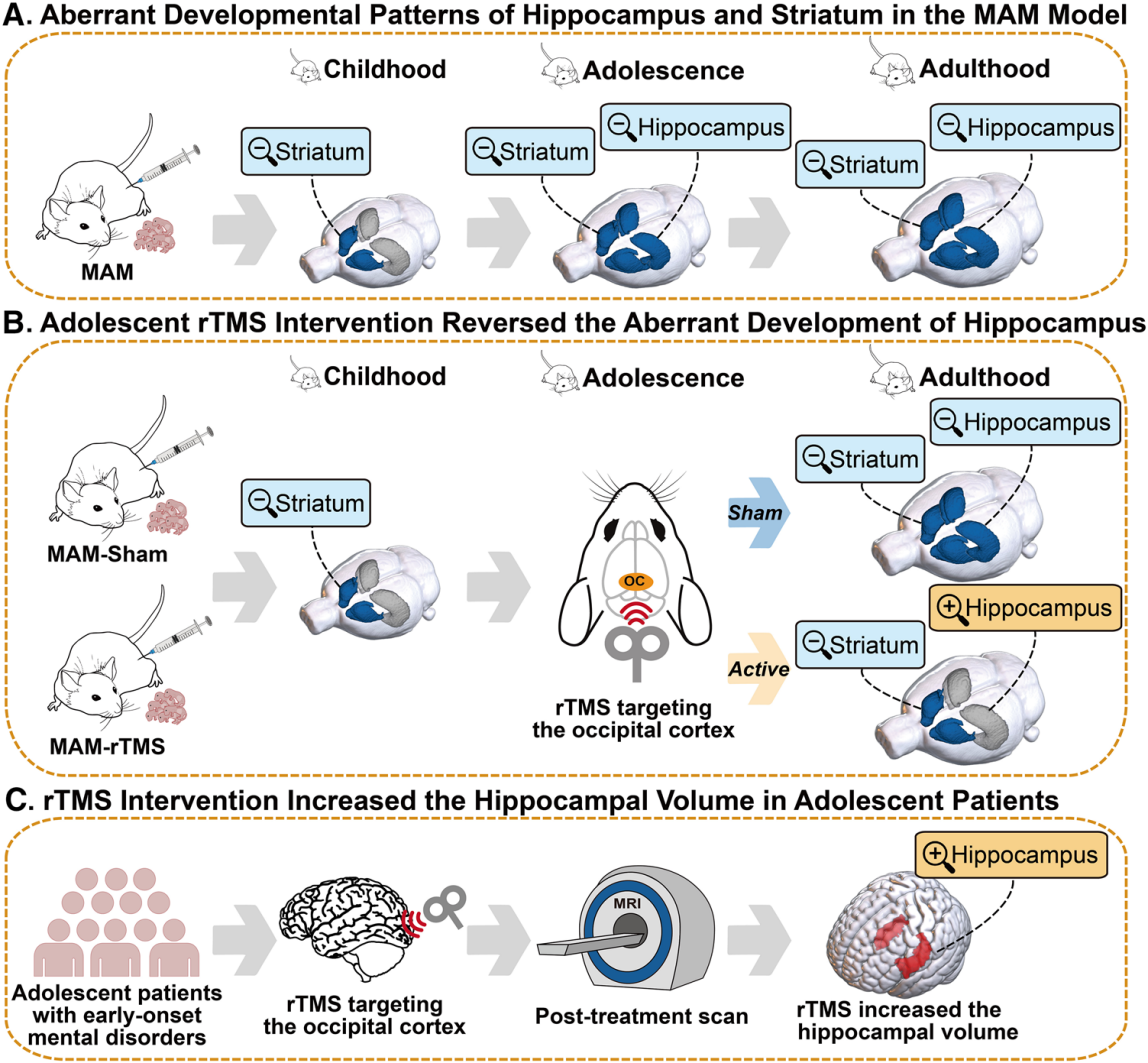
Study overview. **A** In study 1, we established a neurodevelopment animal model, known as the MAM model, and evaluated its brain development patterns over time using longitudinal structural MRI scans. Our findings revealed that delayed volume development of the striatum occurred during childhood, and that of the hippocampus during adolescence, becoming increasingly persistent into adulthood. **B** In study 2, we investigated the impact of occipital rTMS intervention during adolescence on the aberrant development of the hippocampus in the MAM model. Our results showed that this intervention effectively reversed the abnormal development of the hippocampus. **C** In study 3, we verified this intervention paradigm in a prospective cohort of adolescent patients with early-onset mental disorders. Our findings indicated that after 20 sessions of occipital rTMS intervention, the volume of the hippocampus increased. MAM, Methylazoxymethanol acetate; MRI, Magnetic resonance imaging; rTMS, Repetitive transcranial magnetic stimulation

The experiment was divided into three studies (Fig. 1): study 1 was a longitudinal study of brain structural development in the MAM model; study 2 was a cross-sectional study of the effect of adolescent rTMS intervention on the brain structure of the MAM model; and study 3 was an examination of the effect of rTMS intervention on brain structure in adolescent patients with early-onset mental disorders.

To create the animal model, we used MAM, a mitotoxin that interferes with neurogenesis in cortical and subcortical areas when administered at embryonic day 17. In study 1, we investigated the abnormal patterns of gray matter development in the MAM model at different developmental stages, including childhood [postnatal day (PD) 21–25], adolescence (PD 42–46), and adulthood (PD 63–67). The hippocampus and striatum are key regions affected during the abnormal brain development process in the MAM model and have different developmental trajectories. Childhood might be the critical window for intervention in the striatum while adolescence might be the critical window for intervention in the hippocampus. In study 2, we investigated whether rTMS during adolescence affected hippocampal structural development in the MAM model. Given that the target of rTMS is typically limited to the cortex, we selected the occipital cortex, which has a direct neuronal projection to the hippocampus, as the cortical target for rTMS to explore the effects on the hippocampus. We found that this intervention paradigm reversed the abnormal developmental trajectory in the hippocampus but not the striatum of the MAM model. Finally, in study 3, we demonstrated in a prospective cohort that adolescent rTMS targeting the occipital cortex also altered hippocampal structure in adolescent patients with early-onset mental disorders.

### Study 1

#### Animals

All animal procedures were in accordance with the guidelines of the Animal Care and Use of Laboratory Animals and were approved by the Animal Care and Ethics Committee of the Innovation Academy for Precision Measurement Science and Technology, Chinese Academy of Science. Pregnant Sprague–Dawley dams were procured from Vital River (Beijing, China) and randomly assigned to receive either MAM (Wako, Osaka, Japan) at a dose of 22 mg/kg or vehicle (0.9% saline) *via* intraperitoneal injection on gestational day 17 (day 0 was defined as the day when the plug was observed) [35, 36]. In total, 2 MAM pregnant rats and 2 control pregnant rats were used. The pups were weaned 21 days after birth and male pups were kept in cages of three with an unrestricted diet of pellets and water. The animals were housed in a temperature-controlled room (21 ± 2°C) with a 12-h light/dark cycle (lights on from 08:00 to 20:00). A total of 11 control rats and 12 MAM rats were used in the experiment.

#### Animal MRI Data Acquisition and Processing

All animals were scanned at three stages of development (childhood, adolescence, and adulthood) using a 7.0 T Bruker Biospec70/20USR small animal MR system (Bruker, Berlin, Germany). A partial volume transmit coil was used for signal transmission and a surface coil with a diameter of 20 mm was used for signal reception. Each animal was first anesthetized with a mixture of 0.5%–1.0% isoflurane (RWD, Shenzhen, China), 30% O_2_, and 70% N_2_, and their core temperature was maintained at 37°C ± 0.5°C using a warming bed. During the MRI acquisition, the animals were secured in a head restrainer with a built-in coil and a body tube, and their respiratory rate and body temperature were monitored using a PC-SAM Small Animal Monitor (SA Instruments, New York, USA). A T2-weighted structural image was obtained using a fast spin-echo sequence Turbo-Rapid Imaging with Refocused Echoes (RARE) that lasted for 25 min 36 s [repetition time (TR): 6000 ms, echo time (TE): 36 ms, field of view (FOV): 24 mm × 24 mm, slice thickness: 0.4 mm, slice number: 50, matrix size: 256 × 256, resolution: 93.75 μm × 93.75 μm]. Unfortunately, one rat in the MAM group died during the MRI scan.

After completing all MRI scanning, the Bruker ParaVision MRI data were converted to the NIfTI format using Bru2nii [37]. Then, the raw data were manually checked for quality and three data sets from the MAM group were excluded due to poor quality. This resulted in a total of sets of 11 MRI data in the control group and 9 in the MAM group undergoing subsequent data processing. The remaining data were segmented into gray matter (GM), white matter (WM), and cerebrospinal fluid (CSF) probability maps using the Statistical Parametric Mapping 12 (SPM12) (https://www.fil.ion.ucl.ac.uk/spm/software/spm12) and the SIGMA rat anatomical *in vivo* template [38]. Each GM probability map was then resampled into the standard space. All resampled GM images from childhood, adolescence, and adulthood were used to create a study-specific template using diffeomorphic anatomical registration through exponentiated lie algebra (DARTEL) [39]. Each GM image was then warped using the deformation parameters calculated by the DARTEL algorithm and modulated to correct volume changes that occurred during the deformation step. Finally, all normalized GM images were spatially smoothed with a 3 mm full-width-at-half-maximum (FWHM) Gaussian kernel.

### Study 2

#### Animals

The animal model was identical to that used in study 1. We used a total of 4 MAM pregnant rats and 2 control pregnant rats, and we obtained a total of 18 male MAM pups and 10 control male pups. The MAM rats were randomly divided into two groups: the MAM-rTMS group and the MAM-sham group. Both the MAM-sham group and the control-sham group received sham stimulation, while the MAM-rTMS group received active rTMS intervention, with the occipital cortex as the stimulation site. The stimulation protocol was carried out daily for two weeks, beginning in adolescence (PD38–58, including the first 7 days of the adaptation period).

#### Animal rTMS Intervention Paradigm

Before the stimulation protocol, rats were gently placed in a gloved hand and exposed to white noise produced by rTMS application (5 min per day for 7 consecutive days) to adapt to the noise and restraint during the stimulation intervention. The 2-week active/sham stimulation was performed during PD 43–58. A 50 mm air-cooled circular coil (Yiruide Co., Ltd., Wuhan, China) was placed over the rat’s scalp, the coil location being over the visual cortex. The stimulation protocol was as follows: 10 Hz frequency, 6 s on and 15 s off, 900 pulses per session, total duration of 5 min 15 s per session, and 1 session per day for 2 weeks. For the MAM-sham and control-sham group, the same rTMS protocol was applied, but with the coil placed 10 cm laterally above the scalp.

#### Animal MRI Data Acquisition and Processing

After the rTMS intervention, all rats from the three groups were scanned with the same equipment and parameters as in study 1. All MRI raw data were converted to NIfTI format and manually checked. Two sets of MRI data from the MAM-rTMS group were excluded due to poor quality. Therefore, a total of 10 sets of MRI data in the control-sham group, 9 in the MAM-sham group, and 7 in the MAM-rTMS group underwent subsequent processing. For structural MRI data preprocessing, the remaining MRI data were segmented into GM images using the same paradigm as in study 1. All resampled GM images were used to create a study-specific template using the DARTEL algorithm and were warped, modulated, and finally smoothed with a 3 mm FWHM Gaussian kernel.

### Study 3

#### Adolescent Patients With Early-onset Mental Disorders

All procedures followed were in accordance with the ethical standards of the committee on human experimentation and were approved by the Ethics Committee of the Affiliated Nanjing Brain Hospital of Nanjing Medical University. The trial was registered with the Chinese Clinical Trial Registry (ChiCTR2100045391). Informed consent for inclusion in the study was given by all patients and their legal guardians. A total of 20 adolescent inpatients with early-onset mental disorders (MDD and BD) aged 13–18 years were recruited from the Affiliated Nanjing Brain Hospital of Nanjing Medical University. Their onset age ranged from 12 to 18 years. All eligible participants had a diagnosis of an axis I psychiatric disorder (including 11 MDD and 9 BD), assessed through the schedule for affective disorders and schizophrenia for school-age-children present and lifetime version (K-SADS-PL) based on the Diagnostic and Statistical Manual of Mental Disorders, Fourth Edition (DSM-IV) criteria. The exclusion criteria were: major medical comorbidities (e.g., diabetes mellitus, hypertension, and vascular and infectious diseases); neurological disorders (e.g., history of head injury with loss of consciousness for ≥5 min, cerebrovascular diseases, brain tumors, and neurodegenerative diseases); unstable medical conditions (e.g., severe asthma); mental retardation or autism spectrum disorder; contraindications to MRI (e.g., severe claustrophobia, pacemakers, and metal implants); contraindications to rTMS (e.g., metal in head and history of seizures); and current drug/alcohol abuse or dependence. All patients received a stable psychotropic medication regimen throughout the trial.

The severity of clinical symptoms was assessed at pre- and post-rTMS intervention using the Hamilton Depression Rating Scale 17-items (HAMD-17), the Hamilton Anxiety Scale (HAMA), and the Young Mania Rating Scale (YMRS) for quantifying current mood symptoms, and the Brief Psychiatric Rating Scale (BPRS) for quantifying general psychiatric symptoms.

#### Patient rTMS Intervention

After completing baseline clinical symptom assessments and MRI scanning, all participants received 20 sessions of rTMS stimulation to the occipital cortex. The stimulation protocol was as follows: 10 Hz frequency, 100% of the resting motor threshold, 4 s on and 10 s off; 1200 pulses per session, total duration of 6 min 50 s per session, and 2 sessions per day for 10 consecutive days. The rTMS treatment was delivered using a Magneuro 60 magnetic stimulator (Vishee Inc., Nanjing, China) equipped with a figure-of-eight coil device. The precise location of the stimulation target was determined *via* jointly integrating the international 10–10 system and individualized 3D structural MRI. Any self-reported adverse event was recorded after each session of treatment.

#### Patient MRI Data Acquisition and Processing

MRI scanning was performed at baseline and after the rTMS intervention based on the Siemens Magnetom Prisma 3.0 T scanner with a standard 8-channel head coil. A T1-weighted structural image was obtained using a 3D magnetization-prepared rapid acquisition gradient-echo sequence that lasted for 5 min 58 s (TR: 2530 ms, TE: 2.98 ms, FOV: 224 mm × 256 mm, slice thickness: 1 mm, slice number: 192, matrix size: 256 × 256, resolution: 0.5 mm × 0.5 mm × 1.0 mm).

The structural MRI images were processed using Computational Anatomy Toolbox 12 (CAT12) [40] for SPM12. First, all images were manually re-oriented, with the origin point placed at the anterior commissure. Then, all images were segmented into GM, WM, and CSF. The GM images were then spatially normalized to the Montreal Neurological Institute space according to the default CAT12 parameters to obtain images with 1.5-mm^3^ voxels. Finally, all images were smoothed with an isotropic Gaussian kernel of 3-mm FWHM.

### Statistical Analysis

#### Study 1

The GM volume was compared between the control group and the MAM group at three developmental stages using two-way repeated analysis of variance (ANOVA) with a homemade code. Group, time, and the group × time interaction effect were considered significant for voxel *P* <0.05 by false discovery rate (FDR) correction, with a minimum cluster size of 100. We extracted the GM volume for each cluster with significant differences for the group × time interaction and applied the *t*-test to compare the differences between two groups at the same development stage. The significance was set at a threshold of *P* <0.05.

#### Study 2

The GM volume was compared between the control-sham group, the MAM-sham group, and the MAM-rTMS group using one-way ANOVA by a toolbox for Data Processing & Analysis for Brain Imaging (DPABI) [41]. The group effect was considered significant for voxel *P* <0.01 and cluster *P* <0.05 by Gaussian random field (GRF) correction. We extracted the GM volume for each cluster with significant differences among the three groups and applied the *t*-test for pairwise comparisons between any two groups. The significance was set at a threshold of *P* <0.05.

#### Study 3

For the demographic and clinical characteristics, categorical variables are described using frequencies, and continuous variables are presented as the mean ± SD. The clinical characteristics of the subjects pre- and post-rTMS intervention were analyzed by paired *t*-test. GM volume was compared between pre- and post-rTMS intervention using paired *t*-tests by DPABI. The treatment effect was considered significant for voxel *P* <0.05 and cluster *P* <0.05 by GRF correction. We extracted the GM volume for each cluster in the bilateral hippocampus with significant differences and used paired *t*-tests to analyze the differences between pre- and post-rTMS. The significance was set at a threshold of *P* <0.05.

### Compliance with Ethics Requirement

All animal procedures were in accordance with the guidelines of the Animal Care and Use of Laboratory Animals and were approved by the Animal Care Ethics Committee of the Innovation Academy for Precision Measurement Science and Technology, Chinese Academy of Science. All clinical procedures were in accordance with the ethical standards of the committee on human experimentation and were approved by the Ethics Committee of the Affiliated Nanjing Brain Hospital of Nanjing Medical University. The trial was registered with the Chinese Clinical Trial Registry (ChiCTR2100045391). Informed consent for inclusion in the study was obtained from all patients and their legal guardians.

## Results

### Aberrant Development Patterns of the Hippocampus and Striatum in the MAM Model

**Fig. 2:**
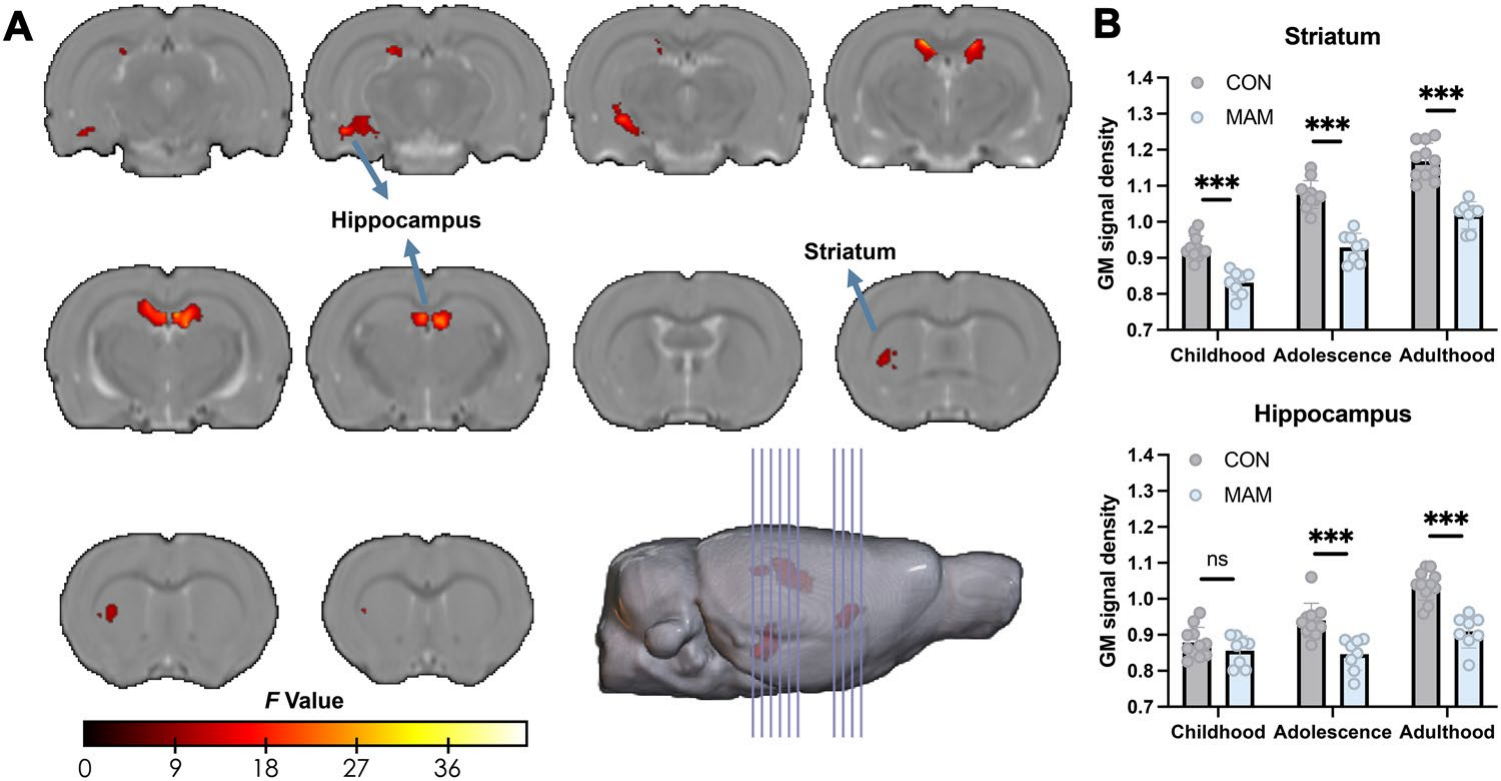
A longitudinal study of brain structure development in the MAM model. **A** Two brain regions, the striatum and hippocampus, were identified using two-way repeated ANOVA with two groups (CON, *n* = 11; MAM, *n* = 9) and three time stages (childhood, adolescence, and adulthood). The MRI data were corrected by FDR with voxel *P* <0.05 and cluster size >100. **B** Using the *t*-test on the extracted GM volume between the CON and MAM groups in the same time stage revealed that the decrease in volume of the striatum occurred from childhood and aggressively persisted through all three time stages (*P* <0.001). The decrease in the volume of the hippocampus occurred from adolescence and aggressively persisted into adulthood (childhood, *P* >0.05; adolescence, *P* <0.001; adulthood, *P* <0.001). The measurement data are presented as the mean ± SD. The significance was set at a threshold of *P* <0.05. ns represents *P* >0.05; *P <0.05; **P <0.01; ***P <0.001. MAM, Methylazoxymethanol acetate; ANOVA, Analysis of variance; CON, Control; FDR, False discoveryrate; GM, Gray matter

Compared to the control group during the three development stages, the MAM group showed significantly delayed development in the GM volume of most brain regions from childhood to adulthood (Fig. S1). Significant time effects were observed in the cortical margins and hippocampus between the control and the MAM groups (Fig. S2). As for the group × time interaction effect, significant clusters were found in the striatum and hippocampus (Fig. 2A). The detailed cluster information is shown in Table S1. GM volume extraction analysis found that the MAM group exhibited developmental patterns in the striatum and hippocampus different from the control group. In the MAM group, abnormality in the development of the striatum persisted from childhood to adulthood (all *P-*values for childhood, adolescence, and adulthood <0.001) and continued to worsen (Fig. 2B). As for the hippocampus, there was no significant group difference in childhood (*P* >0.05), but significant group differences were found from adolescence to adulthood (*P* <0.001).

### Adolescent rTMS Intervention Reverses the Aberrant Hippocampal Development

**Fig. 3:**
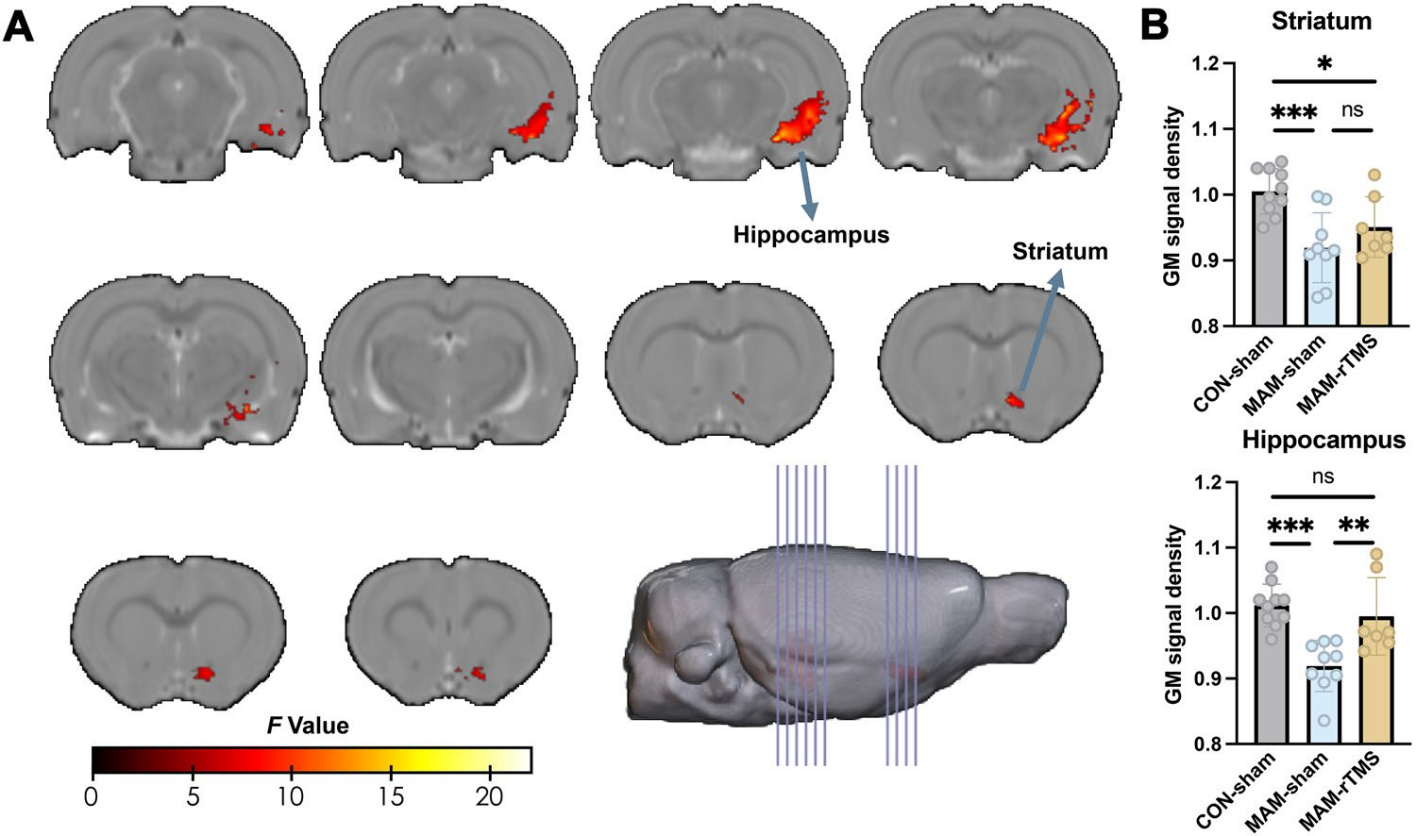
A cross-sectional study of the effect of adolescent rTMS intervention on the brain structure of the MAM model.** A** After the rTMS intervention, two brain regions, the striatum and hippocampus, were identified using one-way ANOVA with three groups (CON-sham, *n* = 10; MAM-sham, *n* = 9; MAM-rTMS, *n* = 7). The MRI data were corrected by GRF with voxel *P* <0.01 and cluster *P* <0.05. **B** Using *t*-tests on the extracted GM volume showed that both the MAM-sham and MAM-rTMS groups had a decreased volume of the striatum compared to the CON-sham group (*P* <0.001 for the MAM-sham group and *P* <0.05 for the MAM-rTMS group). There was no significant difference between the MAM-rTMS group and MAM-sham group (*P* >0.05). The MAM-sham group also showed a decreased volume of the hippocampus compared to the CON-sham group (*P* <0.001). The MAM-rTMS group showed an increased volume of the hippocampus compared to the MAM-sham group (*P* <0.01). There was no significant difference between the MAM-rTMS group and the CON-sham group (*P* >0.05). The measurement data are presented as the mean ± SD. The significance was set at a threshold of *P* <0.05. ns represents *P* >0.05; *P <0.05; **P <0.01; ***P <0.001. *rTMS* Repetitive transcranial magnetic stimulation, *MAM* Methylazoxymethanol acetate, *ANOVA* Analysis of variance, *CON* Control, *GRF* Gaussian random field, *GM* Gray matter.

Significant group effects were also found in the striatum and hippocampus among the control-sham, MAM-sham, and MAM-rTMS groups (Fig. 3A). Detailed cluster information can be found in Table S2. GM volume extraction analysis revealed that the MAM-sham group displayed significantly reduced volume in both the striatum and hippocampus, similar to that found in study 1 (all *P-*values <0.05 between the MAM-sham group and the control-sham group) (Fig. 3B). However, there was no rTMS treatment effect in the striatum (*P* >0.05 between the MAM-rTMS group and the MAM-sham group), but a significant effect in the hippocampus (*P* <0.01 between the MAM-rTMS group and the MAM-sham group).

### rTMS Intervention Increases the Hippocampal Volume in Adolescent Patients

The clinical and demographic characteristics of adolescents with early-onset mental disorders are summarized in Table 1. Clinical symptoms measured by HAMD-17 (*P* <0.001), HAMA (*P* <0.001), YMRS (*P* <0.01), and BPRS (*P* <0.001) changed significantly after the rTMS intervention (Table 2 and Fig. S3).

**Table 1:** Demographic and clinical characteristics

*n* = 20		
	Mean	SD
Age at scan (years)	16.00	1.21
Education (years)	10.25	1.16
Age at onset (years)	14.80	1.88
Duration of illness (years)	1.55	1.55
	*N*	%
Female	12	60
First episode	16	80
Medication		
Antidepressant	1	5
Antipsychotic	19	95
Mood stabilizer	19	95
Sedative hypnotic	11	55

**Table 2:** The effect of rTMS intervention on the clinical symptoms in adolescent patients with early-onset mental disorders

	Pre-rTMS	Post-rTMS
HAMA	21.30 ± 7.86	13.30 ± 6.15
HAMD-17	19.45 ± 5.11	11.05 ± 5.04
YMRS	9.10 ± 5.60	5.95 ± 4.05
BPRS	43.30 ± 10.26	35.00 ± 8.57

Data are presented as the mean ± SD.

HAMA, Hamilton Anxiety Scale; HAMD-17, Hamilton Depression Rating Scale 17-items; YMRS, Young Manic Rating Scale; BPRS, Brief Psychiatric Rating Scale.

**Fig. 4:**
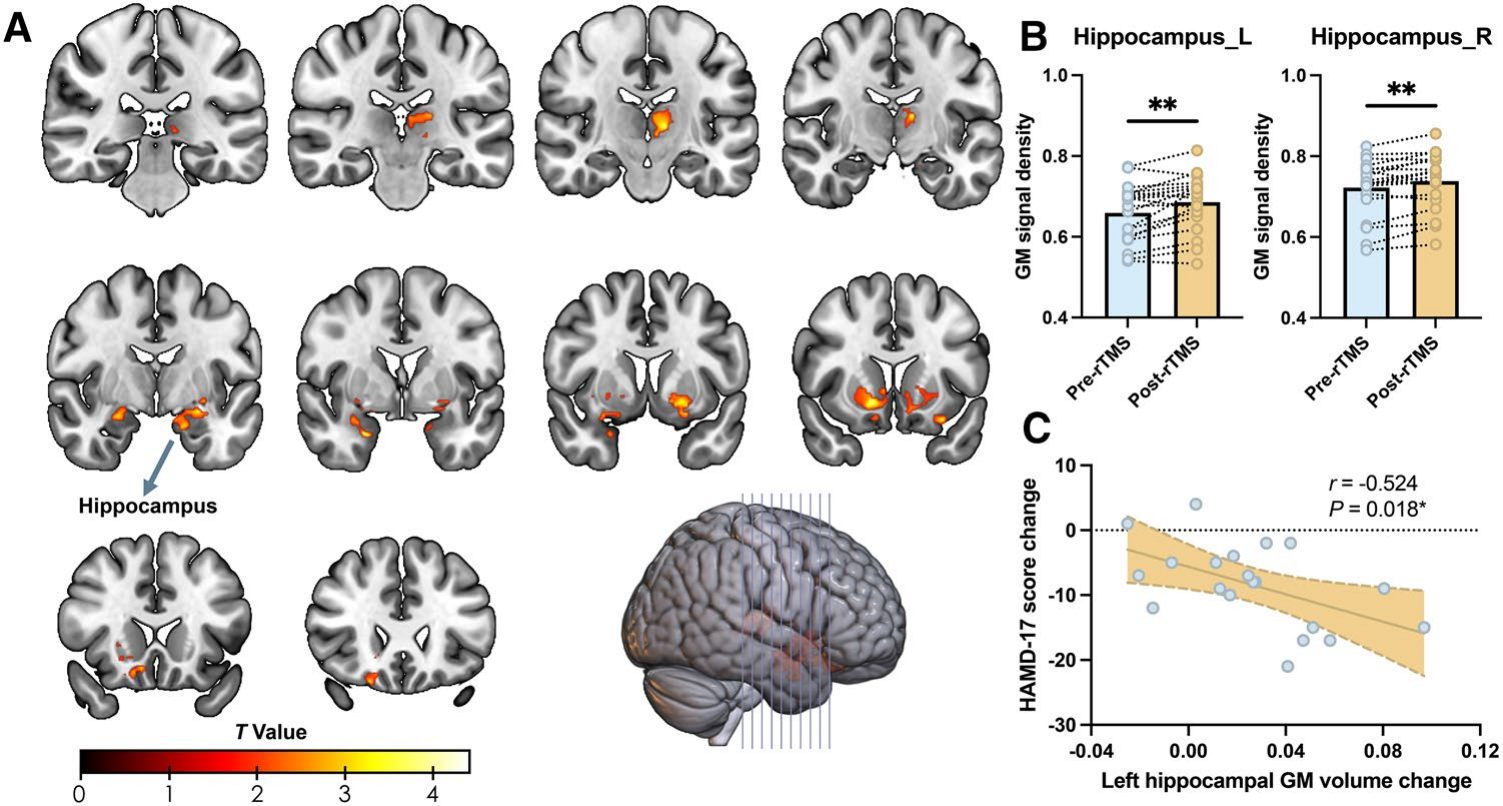
The effect of rTMS intervention on the brain structure in adolescent patients with early-onset mental disorders. **A** The GM volume changes between pre- and post-rTMS (*n* = 20) interventions assessed using a paired *t*-test. The MRI data were corrected by GRF with voxel *P* <0.05 and cluster *P* <0.05. **B** Using a paired *t*-test on the extracted GM volume of the bilateral hippocampus showed a significant increase in the volume after rTMS intervention (left hippocampus, *P* <0.01; right hippocampus, *P* <0.01). **C** Pearson correlation analyses showed the changed GM volume in the left hippocampus was negatively correlated with the reduction of the HAMD-17 score (*r* = − 0.524, *P* <0.05). The measurements are presented as scattered points. The significance was set at a threshold of *P* <0.05. ns represents *P* >0.05; *P <0.05; **P <0.01; ***P <0.001. *rTMS* Repetitive transcranial magnetic stimulation, *GM* Gray matter, *GRF* Gaussian random field, *HAMD-17* Hamilton depression rating scale-17 items.

A significant rTMS treatment effect was found in the bilateral hippocampus (Fig. 4A). Detailed cluster information can be found in Table S3. GM volume extraction analysis revealed that the volume of the bilateral hippocampus increased after the rTMS intervention in these adolescent patients (left hippocampus, *P* <0.01; right hippocampus, *P* <0.01) (Fig. 4B). In addition, a negative correlation was found between changes in the volume of the left hippocampus and changes in HAMD-17 (*r* = − 0.524, *P* = 0.018)(Fig. 4C).

## Discussion

In this study, we aimed to identify the abnormal trajectory of brain structural development in neurodevelopmental animal models and to identify critical windows of key brain regions for intervention. These findings were subsequently verified in an animal model and adolescent patients with early-onset MDD or BD through cross-species studies. The results suggest that aberrant development of the hippocampus during adolescence may be a target for early intervention in early-onset mental disorders associated with abnormal neurodevelopment. Adolescent rTMS intervention was found to reverse this aberrant neurodevelopment and also ameliorate the clinical symptoms.

The MAM model showed extensive delayed volume development in most brain regions, but the hippocampus and striatum were the most critical, exhibiting an abnormal development trajectory compared to other regions throughout neurodevelopment from childhood to adulthood. Abnormalities in the hippocampus, such as decreased volume, incomplete development, and cell disarray have been reported in the MAM model and adult patients with early-onset mental disorders [13, 14, 22]. These structural changes are linked to a decrease in cognitive function and may be a symptomatic marker for the early onset of mental disorders, and the decrease in hippocampal volume could potentially be a neural marker associated with these disorders [42, 43]. By exploring the longitudinal development process of the brain structure in the neurodevelopmental animal model, we found that the structural abnormalities of the hippocampus began in adolescence and worsened over time, suggesting adolescence is a critical window for intervention in early-onset mental disorders.

The striatum is crucial for executive and emotional processing, making it a potential target for pathological changes in psychiatric disorders [44, 45]. A smaller caudate nucleus volume has been reported in early-onset mental disorders [46, 47], drug-naïve first episode MDD [48], and adolescents at high risk for mental disorders [49]. However, due to the lack of longitudinal experimental design, the exact period of abnormal striatum development in early-onset patients with mental disorders remains unknown. Our study in a neurodevelopmental animal model found that abnormal striatum development may start in childhood and progress until adulthood. Because of the early onset of structural abnormalities and the difficulty in identifying clinical manifestations during this period, early intervention targeting the striatum may be challenging to translate into clinical practice. Therefore, in the subsequent experiments, we focused on exploring if adolescent-stage intervention in the neurodevelopment animal model can reverse abnormal hippocampal development.

Subsequent experiments showed that adolescent rTMS intervention in the neurodevelopmental animal model reversed the abnormal developmental trajectory of the hippocampus but not the striatum, consistent with our previous speculation. A prospective study in adolescent patients with early-onset mental disorders provided further evidence for the impact of adolescent rTMS on hippocampal structure. The mechanism by which rTMS affects brain activity is unclear, with limited studies in the developing brain. It is thought to affect synaptic plasticity [50, 51]. Adolescence is marked by heightened levels of lifelong hippocampal neurogenesis, crucial for emotion regulation and cognitive function [52, 53]. Embryonic neurodevelopmental disturbances impact hippocampal neurogenesis and lead to subsequent abnormalities in hippocampal development, possibly related to a deficit in synapse plasticity and accelerated synapse pruning [54, 55]. Therefore, we speculate that adolescent rTMS intervention may regulate the synaptic pruning process to restore the normal hippocampal development trajectory. Further research is needed to confirm this.

Another interesting finding of this study is that rTMS targeting the cortex can impact the subcortical structure but not the cortex, although it is generally believed the effects of rTMS can only reach the cortical area. We chose the occipital cortex for rTMS intervention to study its effect on the hippocampus, which has a direct anatomical connection with the occipital cortex [56, 57]. Previous studies have demonstrated that activity in the occipital cortex can influence hippocampal plasticity [58]. Therefore, we hypothesized that stimulating the occipital cortex using rTMS can lead to changes in hippocampal structure. In the MAM model, we found that adolescent rTMS intervention targeting the occipital cortex reversed abnormal hippocampus development. which was further verified in the cross-species studies in a patient cohort. Animal studies have shown that high-frequency rTMS can induce persistent molecular changes associated with neural plasticity in the cortex and hippocampus [59], indicating that rTMS can not only affect cortical regions but also induce neuronal plasticity changes in subcortical areas. Some studies investigated the impact of rTMS on brain structure, particularly in subcortical regions distant from the cortical target. Research has found that high-frequency rTMS stimulation of the dorsal lateral prefrontal cortex can increase the volume of the amygdala [60], hippocampus [61], and bilateral thalamus [62] in patients with psychiatric disorders, consistent with our findings. This suggests that rTMS intervention targeting the cortex can lead to changes in subcortical structures, possibly due to anatomical connections between the cortex and subcortical regions. We also found that the improvement in depressive symptoms after rTMS was linked to changes in hippocampal volume. This highlights the importance of early intervention for adolescent mental disorders and the potential of adolescent-focused interventions to alter disease progression.

There are still some limitations in this study. First, in animal experiments, anesthesia is typically administered to minimize head movement and stress during MRI scans. Therefore, it is important to consider the potential effects of anesthesia on the experimental results. Second, medication may influence the effectiveness of rTMS intervention. While short-term medication use may not have significant effects on brain structure, caution should still be exercised when interpreting our conclusions. Third, gender may also impact the experimental results. As our study was a preliminary clinical trial, future research with a larger sample size is needed to investigate the potential effects of gender. Fourth, we did not explore the molecular mechanism and intervention effect of rTMS on abnormal neurodevelopment. Future studies should focus on exploring the molecular biological mechanisms during this process. Fifth, due to the current limitations of rTMS stimulation targeting the cortex, future endeavors may aim to use rTMS coils or transcranial-focused ultrasound that can directly stimulate deep brain nuclei to target the hippocampus and assess its impact on neurodevelopment. Finally, the absence of a healthy adolescent control group makes it difficult to determine the abnormal hippocampal structure in adolescent patients with early-onset mental disorders in our study, but previous studies have shown reduced hippocampal volume in these patients. Therefore, this study collected data from 20 adolescent patients for a prospective investigation to explore the effects of rTMS on the hippocampus.

In conclusion, key brain regions and a critical window for early intervention of early-onset mental disorders were identified for the first time in a neurodevelopmental animal model based on a longitudinal research design. Adolescence may be the crucial time to intervene in hippocampal maldevelopment in early-onset mental disorders. Cross-species research with structural MRI in animal models and adolescent patients has further demonstrated that rTMS intervention during adolescence can reverse abnormal neurodevelopmental patterns in the hippocampus. In addition, we also found that rTMS can affect structural changes in subcortical areas.

### Supplementary Information

Below is the link to the electronic supplementary material.

Supplementary file1 (PDF 461 KB)
