# Supplementary material for: Aberrant Hippocampal Development in Early-onset Mental Disorders and Promising Interventions: Evidence from a Translational Study
Source: Neurosci Bull. 2023 Dec 23;40(6):683–94. doi: 10.1007/s12264-023-01162-2 (PMC11178726; doi:10.1007/s12264-023-01162-2)
Supplement: Supplementary file 1 — Supplementary file1 (PDF 461 KB) [file 12264_2023_1162_MOESM1_ESM.pdf]

Supplementary Materials

Supplementary Figures

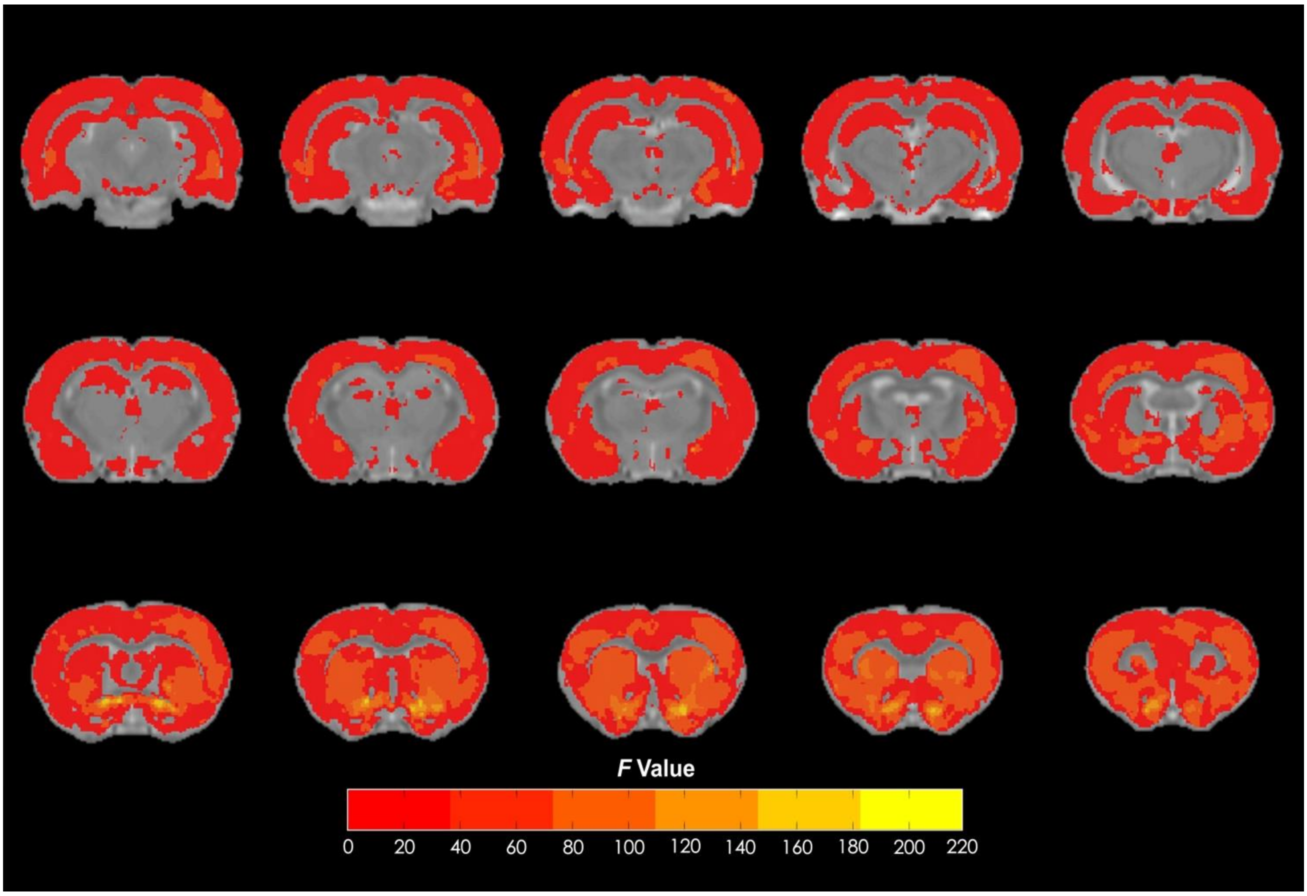

Fig. S1 The MAM model shows extensive gray matter dysplasia during neurodevelopment compared to the control group. The group effect results of two-way repeated ANOVA with two groups (MAM and CON) and three time periods (childhood, adolescence, and adulthood). The MRI data are corrected by FDR with voxel *P* <0.05 and cluster size >100. MAM, methylazoxymethanol acetate; CON, control; ANOVA, analysis of variance; FDR, false discovery rate.

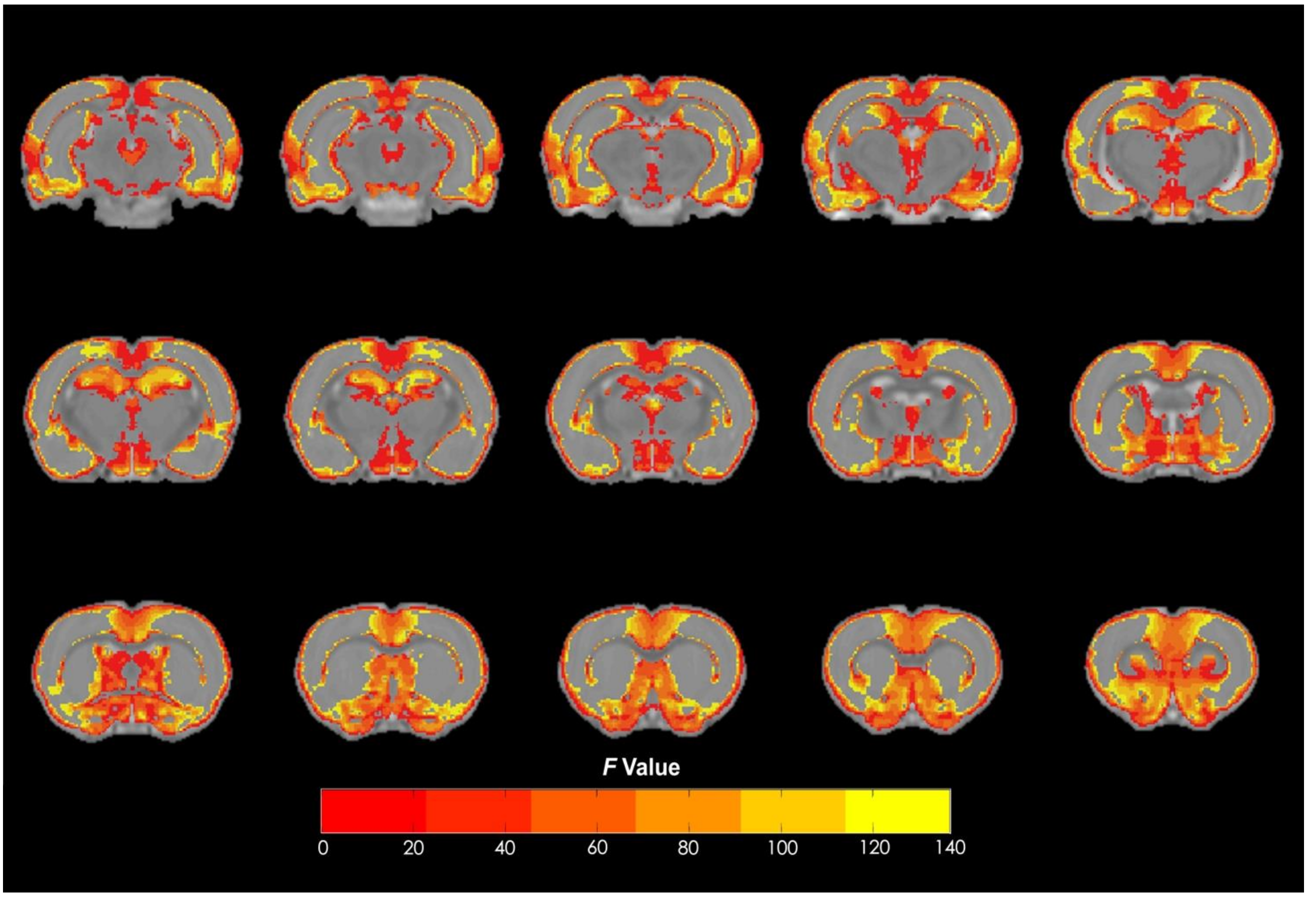

Fig. S2 Gray matter volume changes with neurodevelopment in both the MAM and CON groups. The time effect results of two-way repeated ANOVA with two groups (MAM and CON) and three time periods (childhood, adolescence, and adulthood). Both groups show changes in gray matter volume mainly in the edge of the cortex and some subcortical regions. The MRI data are corrected by FDR with voxel P <0.05 and cluster size >100. MAM, methylazoxymethanol acetate; CON, control; ANOVA, analysis of variance; FDR, false discovery rate.

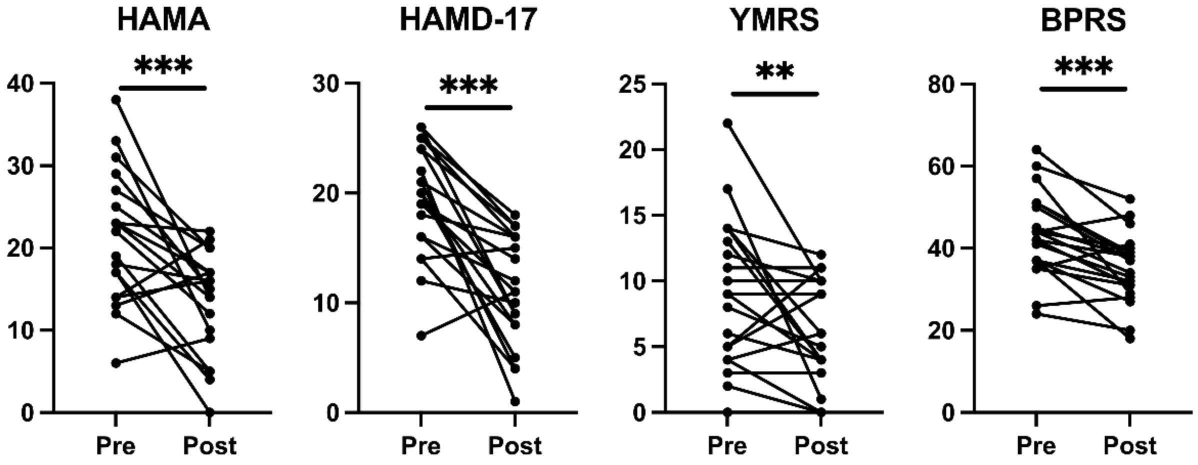

Fig. S3 The change of clinical symptoms pre- and post-20 sessions of occipital rTMS intervention in adolescents with early-onset mental disorders. All four symptom scales show a significant decrease after rTMS intervention (*P* <0.001 for HAMA, HAMD-17, and BPRS, *P* <0.01 for YMRS). The measurements are shown as scattered points. The significance is set at a threshold of *P* <0.05. ns, *P* >0.05; **P* <0.05; ***P* <0.01; ****P* <0.001. HAMA, Hamilton Anxiety Scale; HAMD-17, Hamilton depression rating scale-17 items; YMRS, Young Manic Rating Scale; BPRS, Brief Psychiatric Rating Scale.

Supplementary Tables

Table S1 The cluster information of Study 1

	Cluster size	Brain region	Peak coordinates			Peak intensity
			X	*Y*	*Z*	
Cluster 1	330	Striatum_R	41.90	15.40	16.35	23.003
Cluster 2	1801	CA1<br>DG<br>Fasciola Cinereum	7.40	−38.60	41.85	45.923
Cluster 3	123	Subiculum_R	23.90	−52.10	41.85	15.131
Cluster 4	703	Subiculum_R<br>DG_R<br>CA1_R<br>CA3_R	55.40	−53.60	−10.65	26.144

Four clusters (mainly in the striatum and hippocampus) were identified using two-way repeated ANOVA with two groups (MAM and CON) and three time periods (childhood, adolescence, and adulthood). MAM, methylazoxymethanol acetate; CON, control; CA, cornu ammonis; DG, dentate gyrus; R, right.

Table S2 The cluster information of Study 2

	Cluster size	Brain region	Peak coordinates			Peak intensity
			X	*Y*	*Z*	
Cluster 1	420	Striatum_L	−12.10	10.90	−7.65	11.890
Cluster 2	2663	Subiculum_L<br>DG_L<br>CA1_L<br>CA3_L	−43.60	−41.60	−12.15	23.668

Two clusters (mainly in the striatum and hippocampus) were identified after the rTMS intervention using one-way ANOVA with three groups (MAM-rTMS, MAM-Sham, and CON-sham). MAM, methylazoxymethanol acetate; rTMS, repetitive transcranial magnetic stimulation; ANOVA, analysis of variance; CON, control; CA, cornu ammonis; DG, dentate gyrus; L, left.

Table S3 The cluster information of Study 3

	Cluster size	Brain region	Peak coordinates			Peak intensity
			X	*Y*	*Z*	
Cluster 1	808	Putamen_L<br>Hippocampus_L<br>Amygdala_L<br>Caudate_L<br>Insula_L	−21	9	−13.5	4.071
Cluster 2	1318	Putamen_R<br>Amygdala_R<br>Caudate_R<br>Olfactory_R<br>ParaHippocampal_R<br>Hippocampus_R	24	1.5	−27	5.421

Two clusters (mainly in the bilateral hippocampus) were identified by paired *t*-tests of pre and post rTMS intervention. rTMS, repetitive transcranial magnetic stimulation; CA, cornu ammonis; DG, dentate gyrus; L, left; R, right.
